# Supplementary material for: Pulsed Broad-Spectrum UV Light Effectively Inactivates SARS-CoV-2 on Multiple Surfaces and N95 Material
Source: Viruses. 2021 Mar 11;13(3):460. doi: 10.3390/v13030460 (PMC7998866; doi:10.3390/v13030460)
Supplement: Supplementary file 1 [file viruses-13-00460-s001.pdf]

Supplemental Figures

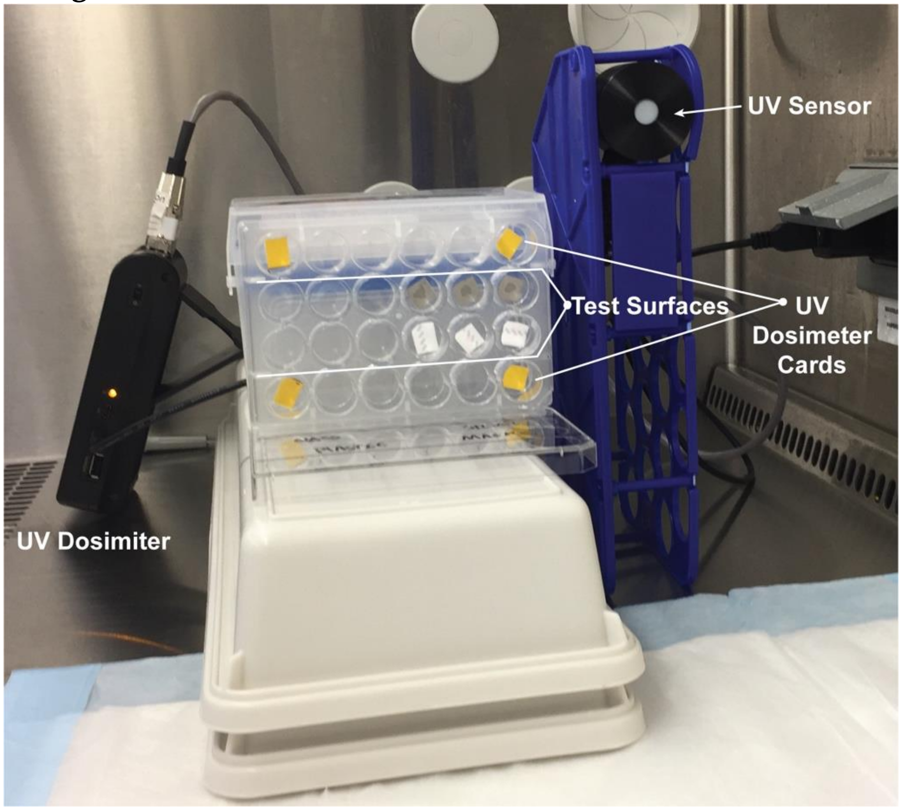

Supplemental Figure S1. Test plate arrangement.

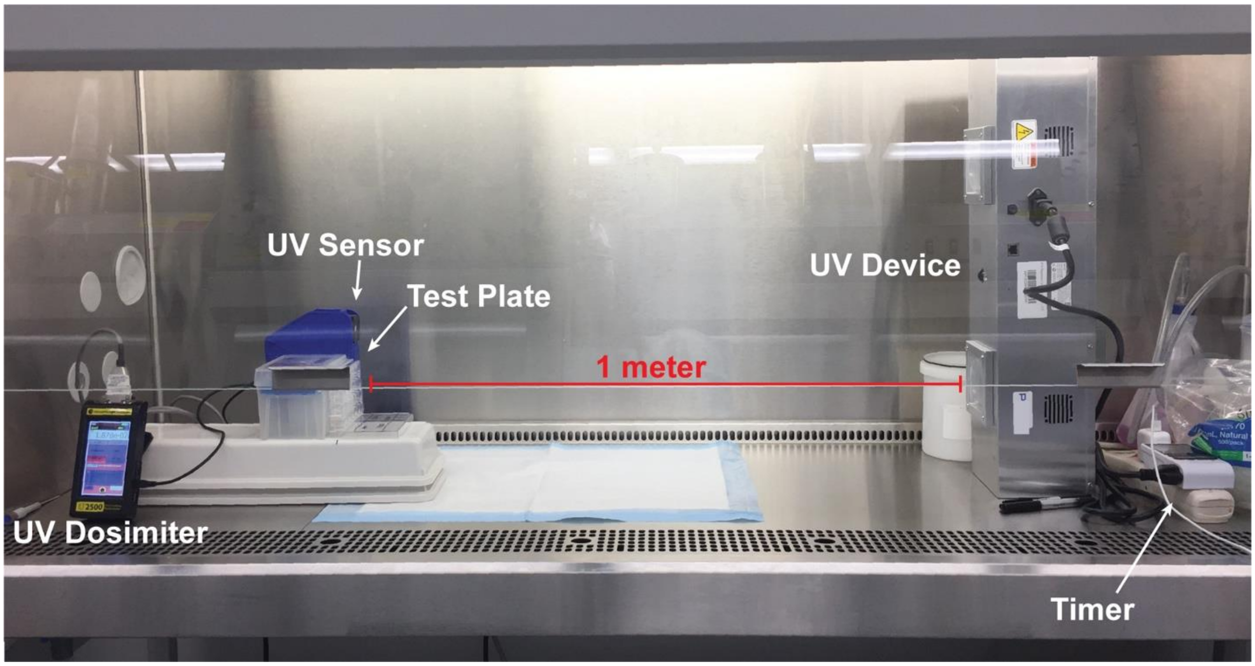

Supplemental Figure S2. Experimental setup.

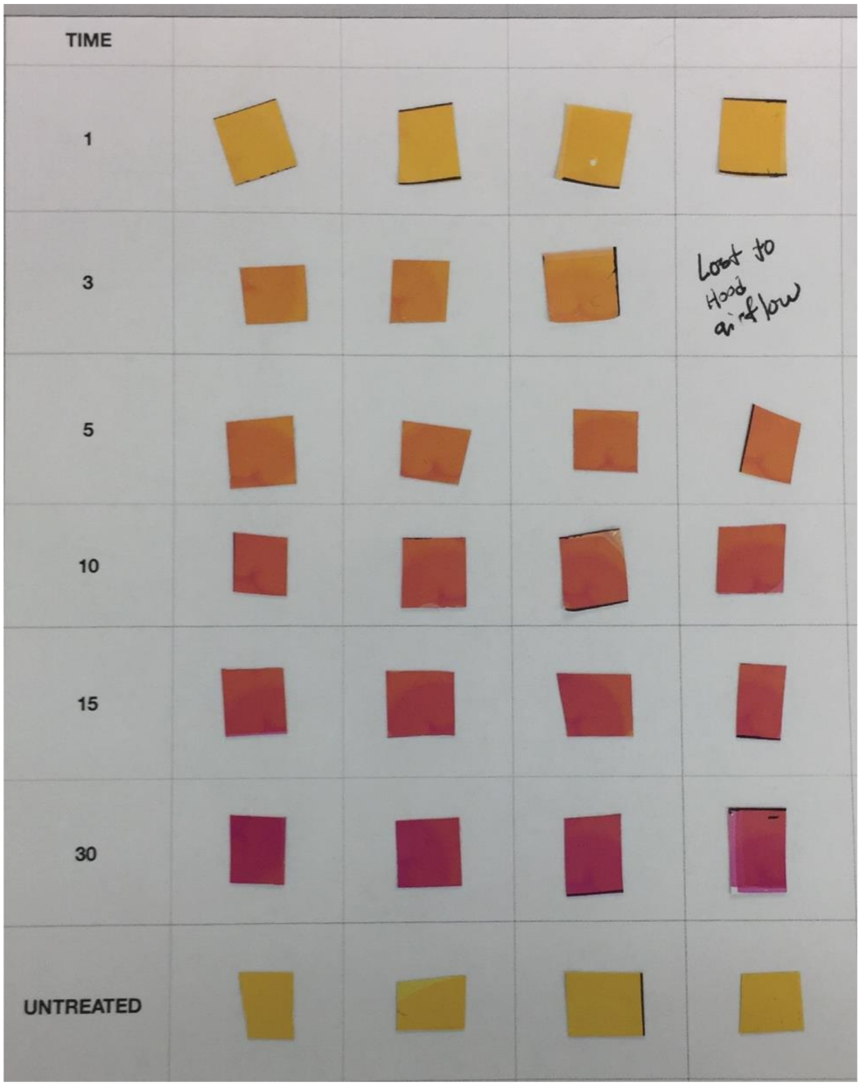

Supplemental Figure S3. UV dosimeter cards collected from each test plate.
